# Implementation of NGS and SNP microarrays in routine forensic practice: opportunities and barriers

**DOI:** 10.1186/s12864-025-11723-6

**Published:** 2025-05-28

**Authors:** Sharlize Pedroza Matute, Sasitaran Iyavoo

**Affiliations:** 1 School of Natural Sciences, University of Lincoln, Brayford Pool, Lincoln, LN6 7TS UK; 2 AttoGroup Limited, Scottow Enterprise Park, Badersfield, Norwich, NR10 5FB UK

**Keywords:** Next generation sequencing (NGS), Massively parallel sequencing (MPS), DNA microarrays, Short tandem repeat (STR), Single nucleotide polymorphism (SNP), Forensic genetics, Forensic DNA phenotyping (FDP), Forensic investigative genetic genealogy (FIGG), Kinship testing

## Abstract

Forensic DNA analysis plays a pivotal role in personal identification, kinship assessment, and criminal investigations, with Short Tandem Repeat (STR) typing via capillary electrophoresis (CE) long established as the gold standard. However, CE-based STR analysis faces notable limitations in multiplexing capacity, the interpretation of degraded or mixed samples, and the resolution of complex kinship relationships. Emerging technologies such as Next Generation Sequencing (NGS) and Single Nucleotide Polymorphism (SNP) microarrays present promising alternatives that can address these shortcomings and expand the scope of forensic DNA testing. Despite their potential, the adoption of these methods in routine forensic practice remains limited due to high costs, technical complexity, and a lack of standardised protocols and legal frameworks. This review critically examines the capabilities, limitations, and current applications of NGS and SNP microarrays in comparison to traditional STR CE profiling. NGS enables STR sequencing and SNP typing with enhanced discriminatory power, better performance with degraded DNA, and improved mixture deconvolution. Conversely, SNP microarrays offer a cost-effective solution for extended kinship testing, Forensic Investigative Genetic Genealogy (FIGG), and phenotypic prediction, though they are less effective with low-quality samples and DNA mixtures. Ethical, legal, and privacy concerns, particularly surrounding the use of Forensic DNA Phenotyping (FDP) and consumer genetic data in FIGG, further complicate their integration into forensic workflows. While significant challenges remain, technological advancements and growing regulatory efforts point towards an achievable path for wider implementation. A hybrid approach that combines STR CE for routine casework with NGS and SNP microarrays for complex scenarios, supported by investments in bioinformatics training, database expansion, and ethical governance, offers a practical strategy for integrating these technologies into future forensic practice.

## Background

DNA analysis was introduced into forensic practice around 40 years ago, providing powerful new evidence and revolutionising the criminal investigation process. The first method employed was DNA fingerprinting using Restriction Fragment Length Polymorphism (RFLP). Although innovative, this technique, which analysed polymorphic DNA regions of variable length called Variable Number Tandem Repeats (VNTRs), was lengthy and labour-intensive. Thanks to the discovery of Polymerase Chain Reaction (PCR), the use of RFLP was quickly surpassed by a more efficient technology based on the amplification of STR DNA regions and fragment separation via CE [1, 2]. This subsequently became the most popular DNA profiling method used in forensic genetics, with commercial kits being developed alongside standardised operational and statistical methods for analysis and interpretation [3]. 

To further support criminal investigations, countries across the world began developing national DNA databases with the aim of linking suspects to past and future cases or exonerating them [4, 5]. Despite its success and widespread use, STR typing via CE faces some limitations, including restricted multiplexing capabilities (i.e. the number of loci that can be typed simultaneously), reduced success with low-quality and low-quantity DNA, and limited power in discerning individual profiles in mixtures. 

To overcome these limitations, additional technologies and alternative types of markers have been explored for forensic use. Among these, NGS and the analysis of differences in STR sequences, as well as the detection of variation in single DNA bases known as SNPs, have been considered. However, while NGS may provide several advantages over CE STR typing, factors such as prohibitive costs and technical challenges appear to be hindering its routine introduction into forensic practice [6, 7].

Beyond NGS, several other methods are available for SNP testing, including mini-sequencing via microarrays [8]. DNA microarrays are particularly useful for generating large quantities of data, up to hundreds of thousands of markers, at contained costs, offering significant advantages in applications such as kinship testing [9, 10]. SNP data can also provide important investigative leads by allowing the prediction of biogeographic ancestry and phenotypic traits, highlighting the potential of these markers in forensic investigations [11].

Despite these promising developments, NGS and DNA microarrays are still seldom employed in forensic practice. While STR testing via CE may remain the most practical and cost-effective option for routine forensic cases, such as personal identification from good-quality DNA or standard paternity testing, the use of NGS and DNA microarrays in more complex cases could make the difference between solving and not solving a case. This highlights the need to evaluate the barriers delaying their wider introduction.

The aim of this review is to summarise the advantages and disadvantages of NGS methods and DNA microarrays for STR and SNP typing compared to STR CE testing, to highlight the factors limiting the adoption of these newer technologies (including legal and ethical concerns), and to provide a series of recommendations to promote their integration as forensic tools in routine casework. In doing so, we aim to draw attention to the valuable contributions that NGS and DNA microarrays can offer to forensic genetics and to encourage researchers and forensic professionals to work towards addressing the barriers preventing their widespread adoption.

## Current techniques in forensic DNA analysis

### The gold-standard: STR typing via CE

#### STR characteristics and CE

STRs, also known as microsatellites, are DNA regions consisting of short sequences (1–6 nucleotides) repeated up to 100 times in tandem. While certain STRs feature identical repeat units, others may show added variation due to inconsistencies in their sequences. STRs are the most abundant tandem repeat sequences in the human genome, totalling between 700,000 and 1,000,000. Of these, only around 8% are located in coding regions, leading to a high number of available markers with minimal linkage to human traits.

The mutation rate of these markers lies between 10^− 6^ and 10^− 2^ per generation, producing differences between ancestors and descendants that enable individual differentiation. Mutations cause variation in both the STR sequence (sequence polymorphism) and the number of repeated units (length polymorphism), the latter likely due to slippage events during DNA replication [12–15].

The advantage of length polymorphism lies in the ability to separate alleles by size. STR markers can be amplified using PCR and labelled with different fluorescent dyes to enable size separation via capillary gel electrophoresis. This method enables fast and reliable typing, whereby the length of each fragment corresponds to the number of repeats present in each allele, and the signal is proportional to the initial amount of DNA present in the sample. By combining different but compatible primer sets in the PCR reaction, results from multiple markers can be generated simultaneously. The multiplexing limit is defined by marker size and the number of dyes that can be distinguished in a single run without spectral overlap [16, 17].

Ideal STR markers should exhibit high heterozygosity, short overall sizes, low stutter artefact levels, and relatively low mutation rates to minimise complications in paternity testing [18].

#### Commercial kits and databases

To facilitate testing and standardisation, several companies have developed commercial kits with pre-mixed primers and all components necessary to type selected loci consistently. The selection of core STR loci, such as the Combined DNA Index System (CODIS) loci by the FBI’s National DNA Index System (NDIS) [19], and the European Standard Set (ESS) by the European Network of Forensic Science Institutes (ENFSI) [20], has enabled the establishment of DNA profile databases and promoted data sharing, contributing to the success of STR typing.

The United Kingdom was the first country to establish a National DNA Database (NDNAD) in 1994, which, as of 2024, holds profiles for over 6 million individuals [21, 22]. Similarly, the NDIS currently contains nearly 24 million profiles based on the CODIS core loci [23, 24]. STR profiles have been widely collected across the globe, with population-specific databases established to provide allele frequency data for multiple populations, enabling precise statistical calculations [17, 25]. Additionally, numerous guidelines have been produced to establish frameworks for comprehensive forensic validation and laboratory practice, promoting the reliability and legal admissibility of STR-based forensic genetics reports [26].

Popular STR kits, such as the GlobalFiler PCR Amplification Kit (Thermo Fisher Scientific) [27] and the PowerPlex Fusion 6C System (Promega) [28, 29], use six dyes and just over 20 markers, producing probabilities of identity ranging from 3.09 × 10^− 26^ to 9.09 × 10^− 31^ across various population groups. The number of markers that can be processed simultaneously using CE is limited by the number of dyes in the assay. However, efforts to expand beyond the standard six dyes to eight have been reported. For instance, the PowerPlex 35GY System (Promega) enables the analysis of 35 loci, including 15 mini-STRs, loci shorter than 250 nucleotides that are particularly useful in degraded DNA samples [30]. Whereas most STR kits include markers with fragment sizes between 100 and 450 bases, the use of mini-STRs improves performance with degraded samples. Degradation typically affects longer fragments first, resulting in profiles with gradually lower peak heights for larger loci [31].

#### CE STR typing limitations

While STR typing has established itself as the most successful forensic DNA typing method, relatively inexpensive, fast, and capable of high discrimination between individuals, several critical challenges remain unresolved [3]. These include difficulties when dealing with challenging samples such as low-template DNA, degraded DNA, and mixtures. 

STR kit reagents, protocols, and practices have been optimised to improve the likelihood of obtaining usable profiles from low-quality or low-quantity DNA samples. This includes the use of consensus profiles, increased PCR cycle numbers, and the implementation of mini-STRs. Although these methods mitigate stochastic effects such as allelic drop-out and drop-in, the potential to reduce STR size further is limited by the nature of the marker itself, thereby restricting the testing of heavily degraded samples, for instance, in mass disaster scenarios. Moreover, the limited number of mini-STR markers available may not provide sufficient discrimination power, especially when no comparative profiles or other investigative leads are available [32–34].

Challenges also arise when analysing DNA mixtures from multiple contributors, as is often the case in forensic samples recovered from crime scenes. Despite the development of various analytical approaches and software for mixture deconvolution, detecting minor contributors in electropherograms typically becomes difficult below a 1:19 ratio and is often limited to mixtures involving two individuals. In contrast, other markers and technologies, such as NGS or linked SNPs (microhaplotypes), can offer notable advantages in these situations [34, 35].

Another area where conventional STR testing proves limited is pairwise kinship testing. While commonly used STR markers are highly effective for evaluating first-degree relationships (e.g. parent–child), the failure rate increases for second-degree relationships such as half-siblings or grandparent–grandchild, where the genetic thresholds are less stringent. For third-degree relationships, traditional STRs do not provide adequate discriminatory power, necessitating the inclusion of additional family members or alternative markers such as sex chromosome loci [36]. However, sex chromosome markers are only applicable in specific relationships, such as father–son or male–male siblings, when using Y-chromosomal STRs (Y-STRs) [37]. Autosomal STRs are also less effective in deficiency paternity cases, for example, when the biological mother is not available and mutations are observed between the child and the alleged father. These situations often require additional markers to clarify results [38].

In cases lacking investigative leads, information about a DNA donor’s phenotypic appearance, including likely ancestry, may be valuable. However, this is generally limited when using STRs. These markers are typically located in non-coding DNA regions, decreasing the likelihood of any direct association with observable traits [39]. Historically, STRs were selected primarily for their ability to identify individuals, not for investigative intelligence. While extensive population data have enabled some success in estimating ethnic origin [40, 41], STRs currently offer only limited capabilities in this context, and phenotypic prediction using STRs remains even more difficult. Although future research may reveal STR markers linked to phenotypic traits through linkage disequilibrium, SNPs, which are abundant and evenly distributed across the genome, are increasingly being investigated for such applications [42].

### STR and SNP typing via NGS

#### NGS technology

The first sequencing method developed, known as Sanger sequencing, was a labour-intensive, low-throughput process based on gel electrophoresis. In the early 2000s, this approach was overtaken by NGS technologies, also referred to as Massively Parallel Sequencing (MPS), which dramatically increased the amount of data that could be obtained in a single experiment.

Illumina and Thermo Fisher Scientific are among the most prominent manufacturers of NGS platforms, performing short-read sequencing on millions of individual DNA molecules in parallel. Illumina systems, such as the MiSeq sequencer, use clonal amplification via bridge PCR and detect fluorescently labelled reversible-terminator nucleotides. Thermo Fisher Scientific systems, such as the Ion Torrent and Ion GeneStudio S5, utilise emulsion PCR and detect pH changes associated with nucleotide incorporation [43–45].

**Fig. 1:**
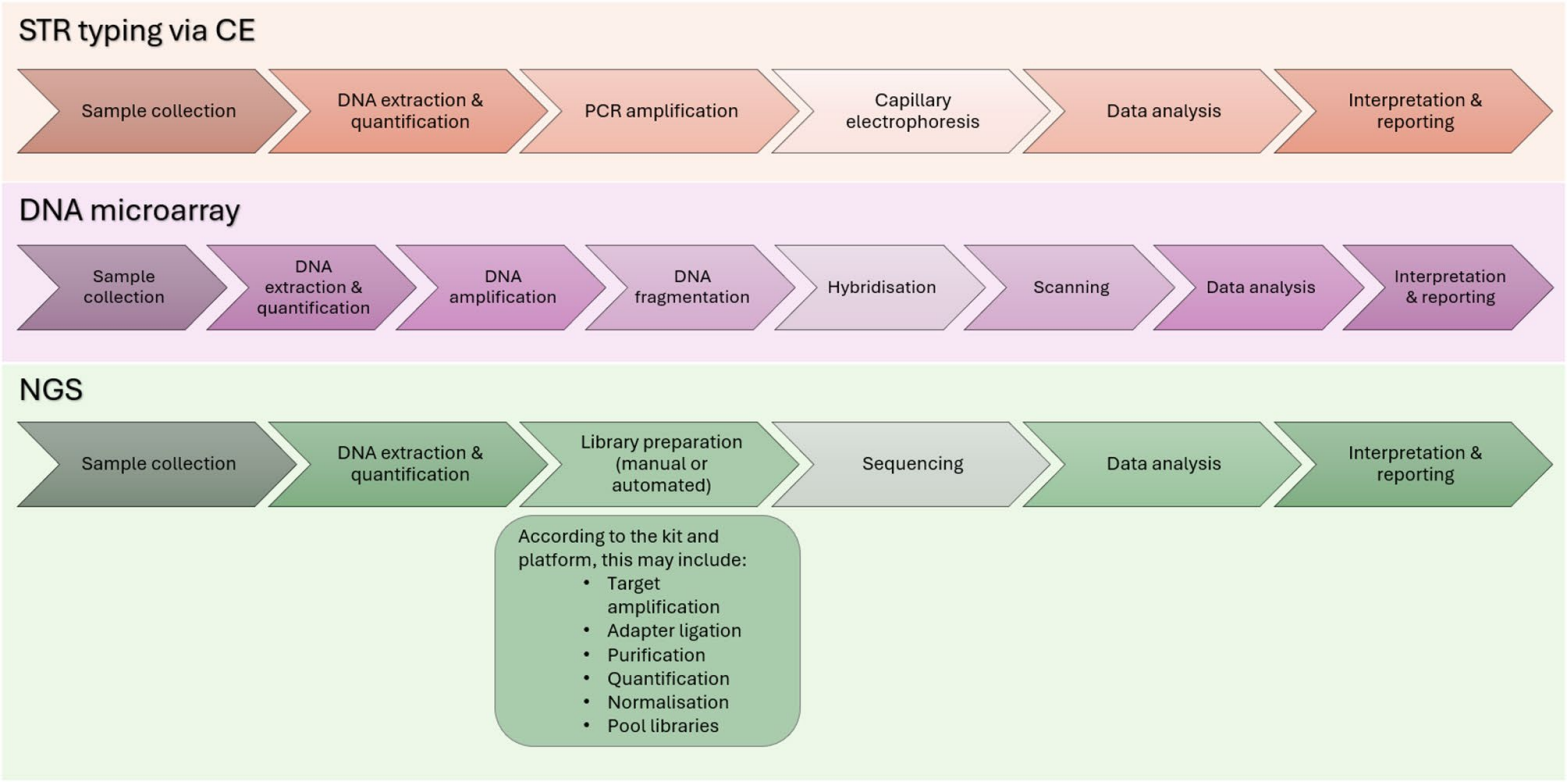
Summary of STR typing via CE, DNA microarray, and NGS technology workflows

A summary of the workflows for STR via CE, DNA microarrays, and NGS technologies is shown in Fig. 1.

Different NGS systems exhibit unique features, each with specific advantages and disadvantages. Illumina technologies are highly accurate but can produce substitution-type miscalls, and they tend to have long run times and higher costs. The Ion Torrent method, although faster and more cost-effective, is less accurate than optical systems and may introduce insertion/deletion (indel) errors, particularly in homopolymeric regions. Greater understanding of platform-specific characteristics and behaviours is essential for developing optimised protocols and bioinformatic tools that minimise errors and improve result accuracy [43, 46, 47].

#### STR sequencing

One of the major advantages of using NGS in forensic science lies in the ability to sequence STR loci directly. By analysing the sequence of each allele, additional polymorphism can be identified. Alleles that are the same length, and therefore indistinguishable by CE, can be differentiated by their sequence, thereby increasing discrimination power [6, 7]. For example, sequencing 26 autosomal STRs in a cohort of 200 White British individuals reduced the Random Match Probability (RMP) by over 700-fold. Including sequence variation in the flanking regions of STRs further reduced the RMP, though to a lesser extent, by approximately fourfold [48].

The increased discrimination power benefits both personal identification and kinship testing. For instance, STR sequencing has proven useful in complex paternity cases by allowing mutation analysis and more accurate interpretation of ambiguous results [49, 50]. Additionally, sequence data can help distinguish true stutter artefacts from alleles of minor contributors in mixtures, and the quantitative nature of sequencing reads aids in evaluating peak ratios. NGS also enables the simultaneous analysis of more loci while maintaining short amplicon sizes, a key advantage for degraded DNA samples, as NGS is not constrained by the dye limits of CE [6, 7, 51].

One of the first commercial MPS kits developed for forensic use was the ForenSeq DNA Signature Prep Kit for the Illumina MiSeq FGx system. This kit includes 27 autosomal STRs, 24 Y-STRs, 7 X-chromosomal STRs (X-STRs), sex-determination markers, and up to 172 SNPs [51]. The ForenSeq system has demonstrated high sensitivity, excellent discrimination, strong reproducibility, and robust performance with challenging samples, including degraded and inhibited DNA [52–55]. Thermo Fisher Scientific also developed the Precision ID GlobalFiler NGS STR Panel, which includes 35 STR markers, covering both CODIS and ESS loci, with 23 shared markers with the CE-based GlobalFiler kit. This panel has shown high sensitivity and good performance with compromised samples, though its mixture analysis results have been inconsistent and do not always outperform CE [56]. Other commercially available STR panels include the Promega PowerSeq Auto System [57] and the Nimagen IDseek OmniSTR Global Autosomal STR Profiling Kit, which uses a simplified reverse complement PCR approach to reduce handling steps during library preparation [58].

Despite the capability of NGS to analyse diverse marker types, the continued inclusion of STR loci in forensic applications remains essential due to the need for compatibility with existing national DNA databases [59]. Consequently, a standardised nomenclature system is necessary for ensuring interoperability between CE and NGS methods and enabling inter-laboratory comparison. To this end, the DNA Commission of the International Society for Forensic Genetics (ISFG) has issued a series of recommendations to address nomenclature challenges [60]. Additionally, the STR Sequencing Project (STRSeq), launched in 2017, has compiled STR sequence variants from approximately 4,600 individuals across various populations to support harmonisation and database development [61].

#### SNP typing

Beyond STRs, NGS enables the analysis of other genetic markers, with SNPs emerging as the most widely studied. In pairwise kinship testing using the ForenSeq DNA Signature Prep Kit, for instance, the addition of SNP data to STR loci has been shown to significantly enhance statistical support for both first- and second-degree relationships [62].

As the most common source of variation between individuals, SNPs are single-base variants occurring roughly once every 1,000 bases, with an estimated total of 4–5 million across the human genome. Unlike STRs, which have relatively high mutation rates, SNPs exhibit lower mutation rates (approximately 10⁻⁸ per nucleotide per generation), making them more stable and suitable for lineage tracing [17, 63].

However, this low mutation rate also limits the number of alleles per locus. While STR loci often exhibit high polymorphism due to numerous alleles, SNPs are primarily biallelic (i.e. they have two alleles), with tri- and tetra-allelic variants being much rarer. As a result, 2.6 biallelic SNPs (with equal 50:50 allele frequencies) are estimated to match the informativeness of one STR locus. To achieve comparable discriminatory power to standard STR kits, between 50 and 100 independent SNPs must be analysed [11, 64]. However, careful selection of rare tri- and tetra-allelic SNPs with high heterozygosity (above 0.5) can improve the power of discrimination [64, 65].

Thanks to the high multiplexing capacity of NGS, the lower individual informativeness of SNPs is not a major limitation. Moreover, the small size of SNPs makes it possible to design very short amplicons, sometimes less than 100 nucleotides, which is advantageous when working with degraded DNA, such as aged remains or environmentally exposed samples [17]. SNP sequencing has also proven more effective than STRs in low-quantity DNA contexts. The higher number of SNPs that can be included in a single assay increases the overall chance of obtaining informative results [66].

Nevertheless, SNP analysis presents challenges, particularly in the detection of contamination and interpretation of DNA mixtures. Due to their biallelic nature, SNPs can produce allelic imbalance and increased heterozygosity in mixtures, making deconvolution difficult [67]. To address this, non-binary SNPs (i.e. tri- or tetra-allelic) and emerging marker systems such as microhaplotypes, short genomic regions containing tightly linked SNPs within ~ 300 nucleotides, have shown great promise in mixture interpretation [68, 69]. A panel of 74 microhaplotypes (230 SNPs), commercially available as the Ion AmpliSeq MH-74 Plex Research Panel, demonstrated very high sensitivity (down to 0.05 ng of DNA) and could reliably detect minor contributors in mixtures down to a 20:1 ratio [70].

In forensic applications, SNPs have been categorised into four main groups [11, 17]:

- Lineage-informative SNPs – useful for tracing ancestral lines (e.g. Y-SNPs);
- Identity-informative SNPs (iiSNPs) – for individual differentiation;
- Ancestry-informative SNPs (aiSNPs) – showing significant inter-population variation;
- Phenotype-informative SNPs (piSNPs) – associated with externally visible characteristics such as hair, eye, and skin colour.

The use of SNPs to predict phenotypic features is referred to as Forensic DNA Phenotyping (FDP). FDP is particularly useful when there are no database matches or eyewitness descriptions, providing investigators with leads that would otherwise be unavailable [71]. However, concerns around accuracy, interpretation, and ethics, especially in jurisdictions lacking legal guidance, continue to limit its use [72].

While the ForenSeq DNA Signature Prep Kit includes iiSNPs, aiSNPs, and piSNPs in addition to STRs, other SNP panels have also been developed. Thermo Fisher Scientific offers:

- The Precision ID Identity Panel, comprising 90 autosomal SNPs and 34 Y-clade SNPs [73];
- The Precision ID Ancestry Panel, with 165 autosomal SNPs for biogeographic inference [74].

Additional panels for phenotypic and ancestry prediction include:

- The VISAGE Basic Tool Research Panel (153 SNPs: 41 piSNPs and 115 aiSNPs) [75];
- The PhenoTrivium Panel, containing 200 autosomal and 120 Y-chromosomal SNPs (Y-SNPs) [76].

Despite the growing number of SNP panels, no universally standardised set of SNP markers has been adopted in forensic practice. This contrasts with STR testing, where CODIS and ESS loci provide standardisation and data-sharing frameworks. As a result, forensic laboratories seeking to implement SNP testing must choose from various panels and kits, which may reduce comparability between laboratories.

#### SNP typing in relationship testing

In addition to their applications in personal identification and the prediction of ancestry and phenotypic traits, SNPs have also proven useful in complex relationship testing. In deficiency paternity cases, for example, the relatively high mutation rate of STRs may result in discrepancies between a child and the alleged father, increasing the risk of misinterpretation, such as erroneously assigning parentage to a relative of the actual father. In such scenarios, the inclusion of SNP testing, characterised by significantly lower mutation rates, has been shown to improve the reliability and statistical strength of results, with approximately 50 biallelic markers offering meaningful support [77].

Although the statistical power of around 50 SNPs may still be lower than that of standard STR kits for parent–child duos or trios, the likelihood of mismatches is considerably lower with SNPs, making them extremely valuable in complex or ambiguous cases [78, 79]. It has been estimated that approximately 85, 127, 491, and 1,858 SNPs are sufficient, respectively, to determine parentage, full-sibling, second-degree, and third-degree relationships. This illustrates the enhanced capability of SNP testing via NGS for comprehensive kinship assessment [80]. Furthermore, discrimination between relatives as distant as second cousins has been reported using as few as 7,000 SNPs [81]. 

Although typing such a high number of SNPs is more readily and economically achieved via DNA microarrays, NGS-based panels have also been developed for extended relationship testing. For example, the FORensic Capture Enrichment (FORCE) panel contains 5,422 SNPs and was specifically designed for long-range kinship analysis. It demonstrated strong statistical support up to the fifth degree of relatedness, and has been successfully used to analyse remains from World War II. The FORCE panel includes a combination of identity-informative, ancestry-informative, and phenotype-informative SNPs, as well as Y-chromosomal and X-chromosomal SNPs, while deliberately excluding clinically relevant variants to avoid ethical complications [82]. Similarly, the ForenSeq Kintelligence kit by Verogen (QIAGEN), containing 10,230 SNPs, was primarily developed for long-range kinship and FIGG. It includes markers for identity, ancestry, and phenotype prediction, and has demonstrated the ability to resolve relationships up to the fourth degree, making it highly suitable for complex forensic investigations [83, 84].

#### NGS limitations

While the advantages of NGS are clear, including aspects not discussed, such as the ability to perform RNA and methylation analysis, identify body fluid types, distinguish between monozygotic twins, sequence mitochondrial DNA, and analyse microbiomes for additional forensic clues [85, 86], several barriers continue to hinder its widespread adoption. A recent survey revealed that, although most European forensic laboratories now possess an MPS platform, its use remains limited. Only a small proportion of laboratories employ dedicated bioinformaticians. Technical challenges, limited training opportunities, lack of standardisation, and insufficient population data for STR sequencing were all identified as areas of concern [87].

Technical limitations of NGS span both laboratory workflows and data interpretation. Compared with CE, NGS protocols are more complex and time-consuming. Additionally, bioinformatic analysis and data interpretation can be challenging, requiring specialised skills and tools [66]. To streamline these processes and minimise the risk of contamination, automated library preparation platforms have been developed, reducing manual handling time. Nonetheless, the high cost per sample, the requirement for specialised personnel, and the need for substantial data storage (due to large raw data files) continue to discourage wider adoption [55, 88].

As the technology advances, promising solutions are being developed to address these limitations. User-friendly software and optimised tools for faster, more accessible data analysis are becoming available. Efforts are also underway to develop nomenclature standards and conversion tools that allow STR sequence data to be translated into CE-compatible formats, ensuring backward compatibility with national databases.

Meanwhile, population studies on STR sequence variation should continue to expand [6]. Although current costs and lengthy protocols (typically 2–3 days) remain limiting factors [59], the ability to combine multiple markers in a single assay, especially those with short amplicons, offers the potential to reduce overall testing time and minimise the need for repeat testing due to failed or inconclusive results. In challenging scenarios, such as degraded samples or distant kinship cases, direct results obtained through NGS may prevent sample depletion from repeated attempts using conventional CE-based methods.

A cost–benefit analysis of large NGS-based SNP panels for FIGG showed that the investment could be more than justified by long-term societal benefits. Reductions in victim numbers and wrongful convictions were projected to result in future savings that far exceed the initial expenditure, supporting the argument for more concrete steps toward implementation [89].

The inherent complexity of NGS and the need to communicate results effectively in court have led to the development of bioinformatic tools such as FDSTools, which assist with analysis and data visualisation. However, additional efforts are required to establish formal guidelines for technical validation, interpretation, and reporting. Furthermore, forensic DNA databases must evolve to accommodate STR sequence data and facilitate its use in routine casework [6, 7].

### SNP typing via DNA microarrays

#### DNA microarrays technology

Compared to NGS, DNA microarrays offer a more cost-effective method for typing Single Nucleotide Variants (SNVs). This technology enables the generation of substantial amounts of data, ranging from thousands to millions of SNPs, in a single assay. DNA microarrays operate through the hybridisation of DNA to short oligonucleotides immobilised on a solid support. Various mechanisms can be employed for genotyping, including allele-specific hybridisation, primer extension, and oligonucleotide ligation, all of which use fluorescence signals to determine allele calls.

The two most commonly used microarray platforms, primarily in clinical whole-genome association studies, are Thermo Fisher Scientific’s Affymetrix arrays and Illumina microarrays. The Affymetrix GeneChip system uses allele-specific probes designed to cover all possible base combinations at polymorphic sites and has been reported in forensic studies, especially for mitochondrial DNA sequencing [8, 90]. The Illumina Infinium platform uses probes complementary to the region of interest, followed by single-base extension with fluorescently labelled nucleotides. This technology has been applied in forensic contexts, particularly in kinship testing and FIGG [91–93].

The high-throughput capability of dense microarrays has enhanced FIGG by enabling the large-scale genotyping of thousands of SNPs per sample. For example, the Infinium Global Screening Array (GSA) includes 654,027 fixed markers and offers customisation with up to 100,000 additional markers. This technology is widely used in direct-to-consumer (DTC) genetic testing services, such as 23andMe, Ancestry, and FamilyTreeDNA, which can identify both close and distant biological relatives (extending beyond first cousins) [94].

The high number of SNPs genotyped also enables the estimation of shared DNA segment lengths, measured in centiMorgans (cM), which can be correlated with degrees of biological relatedness [95]. Genetic results obtained through DTC tests can be uploaded to public databases such as GEDmatch, a genetic genealogy platform that has proven useful in forensic investigations, most notably in the Golden State Killer case, which highlighted the potential of FIGG in resolving cold cases [96].

#### Limitations and opportunities of DNA microarrays in forensics

DNA microarrays offer powerful capabilities for generating high-throughput, accurate SNP data at relatively low cost and with lower technical complexity than NGS, making them particularly advantageous for kinship investigations. Beyond the benefits associated with SNP characteristics already discussed, such as their use in DNA phenotyping and ancestry inference, the ability to design customised panels that include or exclude clinically relevant markers offers additional flexibility and can help address privacy concerns.

Table 1 provides a comparison of the main features of the three forensic DNA technologies discussed in this review.

**Table 1:** Direct comparison of key characteristics of forensic DNA technologies

Feature	STRs via CE	NGS	SNP Microarrays
**Number of markers**	16–35 STRs (limited by dye channels)	Thousands (STRs, SNPs, microhaplotypes)	Hundreds of thousands of SNPs
**Discrimination power**	Moderate (high per marker, low multiplexing)	High (sequence polymorphism and multiple marker types)	High (low per marker, high multiplexing)
**Mutation rate**	Moderate (~ 10^− 6^ to 10^− 2^ per generation)	Varies by marker	Low (~ 10^− 8^ per nucleotide per generation)
**Performance with degraded DNA**	Limited (mini-STRs help)	High (short amplicons allow better recovery)	Moderate (accuracy declines with degraded samples)
**Mixture analysis**	Good (limited for low-level contributors)	High (quantitative read depth supports deconvolution)	Poor (biallelic SNPs limit resolution, but improving)
**Kinship testing**	Good (strong for first-degree)	High (strong for complex and distant relationships)	Very high (suitable up to 4th–5th degree relatives)
**Reliability**	High (well-established and validated)	High (requires standardisation and expertise)	High (validation still developing for forensic use)
**Cost per sample**	Low	High	Moderate
**Turnaround time and complexity**	Fast (~ 6–8 h), low complexity	Slow (~ 2–3 days), high complexity	Slow (~ 2–3 days), lower complexity
**Forensic applicability**	Gold standard for databases and court	Emerging, ideal for degraded DNA and complex cases	Excellent for kinship and genealogy, limited for mixtures

Dense microarrays have been proposed as the future of relationship testing, with the potential to reduce misinterpretations that remain common in STR-based testing. However, forensic validation studies are still limited. More empirical evidence is needed to define their capabilities and limitations, and to establish standardised protocols and interpretation frameworks [97].

The only full forensic validation of the GSA array published to date was conducted by Russell et al. in 2022 [92], while a partial evaluation of a custom Infinium array with over 4,000 markers was reported in 2024 [98]. Both studies helped address one key limitation: the DNA input requirement recommended by the manufacturer. The manufacturer suggests using 200 ng of high-quality DNA, which is considerably more than the 1 ng typically required for other forensic methods. Nevertheless, both studies showed call rates above 99% when using only ~ 1 ng of control DNA, indicating that microarrays can perform well with low-quantity inputs. Similarly, a study by de Vries et al. (2022) [93] demonstrated that 1 ng of high-quality DNA was sufficient for accurate kinship classification up to second cousins using the GSA. Although call rates and genotyping errors slightly increased with lower input, these had minimal impact on relationship inference.

However, when degraded DNA was tested, especially fragments shorter than 150 base pairs, kinship classification success dropped, and genotyping errors, particularly false heterozygous calls, became more common. Naturally degraded samples, such as skeletal remains, often showed lower call rates, whereas casework-like samples (e.g. blood or saliva) provided more reliable results [10, 93]. This suggests that DNA microarrays may be suitable for routine forensic samples, provided that the DNA quantity and level of degradation are properly assessed. However, they may be less reliable for highly degraded materials and more challenging forensic samples.

Similar limitations were noted with Affymetrix arrays when analysing degraded DNA, with elevated false heterozygous calls. A novel analysis method that omitted noise normalisation was developed to improve call accuracy and proved effective in kinship inference [99]. This demonstrates that ongoing research can help develop strategies to overcome current technological constraints. Expanding the range of validated sample types and improving understanding of performance thresholds will allow broader forensic adoption of DNA microarrays, while maintaining awareness of their limitations.

In addition to DNA quality, another challenge is the resolution of DNA mixtures. The biallelic nature of SNPs makes mixture deconvolution inherently difficult, which is one of the primary factors limiting microarray use in forensic settings. Nonetheless, new strategies are emerging. Some methods use allele frequency or intensity data to identify mixtures. For instance, the quantitative nature of intensity signals has been leveraged in a model to detect trace DNA and mixtures involving up to 200 individuals, illustrating the untapped potential of microarrays in forensic mixture analysis [100, 101].

Further opportunities include evaluating which SNPs are more robust with low-quality or low-quantity DNA, and the development of imputation methods. These can infer missing SNP data from available markers, improving comparability between platforms and enabling recovery of partial profiles. Imputation has also been proposed as a way to compensate for mild degradation, with encouraging results for forensic applications [94, 102].

Other limitations, such as the multi-day processing time and the historical lack of proficiency testing for inter-laboratory comparisons, are also being addressed. Technological improvements are gradually reducing processing times, and in 2024, the ISFG English Speaking Working Group launched the first SNP genotyping proficiency trial for forensic laboratories [98, 103].

While challenges remain, ongoing research is steadily improving the technical and practical aspects of DNA microarray use. With continued validation and standardisation, the method holds significant promise for forensic genetics.

## Ethical and legal considerations

### Current standards and regulations

Due to the identifiable nature of DNA data and the sensitive information that may be revealed, including externally visible characteristics, biogeographic ancestry, and potential health predispositions, ethical and legal considerations are particularly important in forensic DNA testing. These considerations vary in scope and complexity depending on the type of genetic analysis conducted.

For routine STR profiling used for personal identification, long-established legal frameworks and ethical standards are in place. The success of STR-based analysis, especially via CE, has prompted many countries to adopt national legislation governing the collection, storage, and comparison of DNA profiles. These are supported by national and international standards, including national DNA databases, accreditation schemes, and legal precedents that ensure the admissibility of STR-based evidence in court [104].

In contrast, the use of MPS and SNP-based forensic methods introduces new ethical, legal, and methodological challenges, depending on the application. Currently available guidance focuses mainly on extending STR applications through MPS. These include:

- Recommendations for STR sequencing and nomenclature by the ISFG [60, 105],
- The National Institute of Justice’s report ‘Implementation Strategies: Next Generation Sequencing for DNA Analysis’ [106],
- STR interpretation guidelines from the Scientific Working Group on DNA Analysis Methods (SWGDAM), along with quality assurance standards from the FBI [107, 108].

In January 2024, SWGDAM also released SNP interpretation guidelines [109], which provide recommendations for NGS-based SNP analysis used in human identification, ancestry inference, phenotypic prediction, and kinship testing. However, these guidelines explicitly exclude whole-genome sequencing, microarrays, and FIGG. Still, they offer valuable recommendations concerning quality assurance, laboratory best practices, and interpretation and reporting principles, all of which are essential for implementing SNP-based NGS in forensic contexts.

Where an application falls outside the scope of these SNP-specific guidelines, broader frameworks such as:

- ISO/IEC 17025 [110],
- ENFSI best practice manuals [111], and
- The UK Forensic Science Regulator’s Code of Practice [112],

can serve as reference points for laboratory compliance, method validation, and operational reliability. However, no detailed regulatory guidance currently exists for many newer applications of forensic genomics.

From a legal standpoint, this regulatory gap creates uncertainty around the admissibility and standardisation of evidence in court. In jurisdictions where precedents or specific legislation are lacking, legal professionals may hesitate to accept results based on new genomic technologies.

On the ethical side, SNP-based methods that infer ancestry or predict phenotypic traits raise significant concerns related to genetic privacy and data protection. These concerns are especially pertinent under the European General Data Protection Regulation (GDPR), which classifies genomic information as sensitive personal data [113]. Forensic applications must also comply with local laws, such as the UK Human Tissue Act, which governs the use and handling of biological materials [114, 115].

Without clear guidelines tailored to advanced forensic genomics, laboratories run the risk of inadvertently breaching individuals’ rights or facing legal challenges. A more comprehensive legal and ethical framework is urgently needed to support the responsible and equitable adoption of these emerging technologies.

### FDP concerns

The practice of FDP is one of the most prominent areas where ethical and privacy concerns arise. SNPs used for phenotype and ancestry prediction are often located within or near genes associated with observable traits. As such, these SNPs are frequently found in coding or regulatory regions of the genome. While FDP can be valuable in generating investigative leads, it may also reveal a predisposition to certain health conditions or sensitive traits such as baldness or behavioural tendencies [71, 116, 117].

Ancestry prediction raises further concerns. It has the potential to lead to racial or ethnic profiling, which could disproportionately target minority communities and contribute to discrimination or bias in investigations [118, 119]. This poses a significant risk of violating fundamental human rights, particularly when combined with the predictive power of FDP and inadequate legal safeguards.

Historically, the success of STR markers in forensic practice has been attributed to their general lack of association with phenotypic traits. For example, the CODIS loci were deliberately selected for their location in non-coding regions and assumed absence of any link to physical or medical characteristics. However, as genetic research progresses, even these markers have come under scrutiny [120]. One example is the TH01 locus, which is located in an intronic region of the Tyrosine Hydroxylase (TH) gene. Specific alleles at this locus have been associated in various studies with traits such as blood pressure regulation, schizophrenia, and other neurological conditions [121]. Although no causal link has been firmly established, the existence of such associations has raised concerns about privacy and the potential misuse or misinterpretation of forensic DNA data.

FDP’s power to generate leads must be weighed against its potential to mislead or misdirect investigations. A notable example occurred in Germany in 2007, in the case of the so-called Phantom of Heilbronn. Authorities pursued a female serial offender based on DNA evidence collected from numerous crime scenes across Europe, including the murder of a police officer. FDP analysis suggested Eastern European ancestry, which influenced investigators to target Sinti and Roma communities. After years of fruitless investigation, it was revealed that the DNA did not belong to a criminal at all, it originated from a Polish factory worker who had unknowingly contaminated cotton swabs during manufacturing. Although the failure was due to contamination and not the FDP analysis itself, the case revealed how FDP-based ancestry predictions can influence the direction of investigations, particularly in the absence of other evidence. It highlighted the importance of strict quality control procedures in forensic supply chains and underscored the risk of racial profiling and investigative bias [122, 123].

As of 2020, only the Netherlands, Germany, and Slovakia had explicitly regulated the use of FDP in forensic investigations. Other countries permitted its theoretical use but lacked legal frameworks specifically governing its application, making the practice a legal grey area. For example, Belgium permits FDP solely for sex determination. In contrast, the United Kingdom allows a broader range of FDP applications, including the prediction of physical appearance, behavioural traits, and medical predispositions, although these are rarely used due to concerns over admissibility in court [124, 125]. The variation in legal approaches across jurisdictions reinforces the urgent need for standardised international guidelines, ethical oversight, and robust quality assurance to ensure that FDP is used responsibly and equitably.

### FIGG concerns

Several ethical and privacy concerns have been raised regarding FIGG since its first high-profile use in the Golden State Killer case. Between the 1970s and 1980s, Joseph James DeAngelo committed a series of violent crimes across California. Although DNA evidence was available, traditional STR typing methods failed to identify a suspect. In 2018, investigators turned to FIGG, uploading the crime scene DNA profile to the public genealogy database GEDmatch, where distant biological relatives were identified. This genealogical connection eventually led to DeAngelo, whose identity was confirmed via STR analysis, culminating in his arrest.

This landmark case showcased the power of DNA microarrays and FIGG in solving decades-old crimes. However, it also prompted significant ethical debate. At the time, GEDmatch users were not explicitly informed that their data could be accessed by law enforcement. This raised questions about informed consent, data privacy, and whether individuals could unknowingly implicate relatives through voluntary participation in consumer genetic testing [126, 127]. As a result, DTC genetic testing companies began restricting access for forensic purposes. Users were subsequently required to opt in if they wished to make their genetic data available for law enforcement use. This shift sparked broader discussions about data ownership, secondary use of personal information, and the need for regulatory oversight in this rapidly evolving field.

In 2019, the United States Department of Justice introduced a policy limiting the use of FIGG to violent criminal cases and restricting searches to databases where users had provided informed consent for law enforcement access. In 2021, Maryland became the first U.S. state to formally regulate FIGG through legislation. This emphasised the importance of balancing genetic privacy, transparency, and public safety, ensuring that DNA data are used responsibly and that individuals’ rights are protected [124, 128].

In Europe, Sweden and Denmark were among the first to adopt FIGG and successfully resolve cold cases. However, implementation across other European countries remains inconsistent. In many jurisdictions, the legality of using consumer DNA databases for investigative purposes is unclear, particularly when considering national data protection laws such as the GDPR. These legal uncertainties are a major limiting factor for FIGG in Europe [129].

In the United Kingdom, FIGG remains a heavily scrutinised and rarely used tool. Although its effectiveness has been demonstrated for individuals of UK ancestry using GEDmatch data [130], law enforcement agencies remain cautious due to ethical and legal challenges. The UK recognises the potential of FIGG to solve crimes and identify disaster victims, but significant concerns around consent, bias, and oversight continue to delay its wider adoption [131]. The UK’s Biometrics and Forensics Ethics Group has recommended the development of a formal ethical and legal framework to support the responsible use of genetic genealogy in criminal investigations. Such a framework would ensure public trust, uphold individual rights, and allow investigators to harness the full potential of genetic data for justice [132].

## Practical recommendations for implementing NGS and SNP microarrays in forensic DNA testing

### Hybrid workflow approach

To successfully incorporate NGS and SNP microarrays into routine forensic casework, laboratories should adopt a hybrid workflow that capitalises on the strengths of each technology. NGS, with its enhanced sensitivity and capacity for multiplexing, is particularly advantageous in complex forensic scenarios, such as highly degraded samples, intricate kinship cases, and DNA mixtures, where traditional CE-based STR typing may be insufficient.

However, due to its higher cost and operational complexity, NGS is not yet a practical replacement for routine forensic profiling. In contrast, SNP microarrays offer a cost-effective and scalable solution for long-range kinship assessments and FIGG, particularly when thousands of markers are required to determine distant biological relationships.

For daily forensic applications, including contributions to national DNA databases, STR typing via CE remains the most efficient and widely accepted method. Therefore, a balanced strategy that:

- employs CE-STRs for routine identification,
- utilises NGS for challenging or unresolved cases, and
- applies SNP microarrays in kinship investigations and FIGG,

will allow laboratories to make the most of technological advancements while maintaining operational efficiency and cost-effectiveness.

### Investment in bioinformatics training

As forensic DNA technologies evolve, bioinformatics training is essential for the effective implementation of NGS and SNP microarrays. Unlike CE-based STR analysis, which relies on well-established, user-friendly software, modern sequencing and microarray workflows generate vast datasets requiring advanced computational processing.

Forensic scientists must be equipped with the skills necessary to:

- interpret complex sequencing results,
- differentiate true genetic variants from sequencing artefacts,
- analyse high-throughput genotyping data, and
- validate outputs for use in legal contexts.

Investment in training programmes, development of forensic-specific bioinformatics tools, and user-friendly automated software will be crucial. These advancements will reduce reliance on external data analysts and support wider accessibility of NGS and microarray technologies in operational casework.

### Gradual database expansion

A major limitation to the widespread adoption of NGS and SNP microarrays is the incompatibility of these technologies with current forensic DNA databases. National databases such as the UK’s NDNAD and the FBI’s CODIS are based on CE-derived STR length polymorphisms, which complicates the integration of sequence-level STR data and SNP-based profiles.

Although NGS enhances resolution by revealing sequence differences between alleles of identical length, the existing STR-based systems already provide strong discriminatory power in most forensic applications. Therefore, a full transition to sequence-level databases may not be economically viable in the short term.

Instead, forensic institutions should pursue a gradual and modular expansion of current databases to:

- accommodate NGS-derived STR sequences alongside traditional CE profiles, and
- begin establishing dedicated SNP databases for kinship and FIGG purposes.

Efforts such as the STRSeq project [61] and ISFG nomenclature standardisation [60] are already paving the way for back-compatibility and inter-laboratory data sharing. Incremental adoption will allow agencies to retain the robustness of established methods while benefiting from the improved capabilities of newer technologies.

### Legal and ethical policy development

Clear and consistent legal and ethical policies are essential to support the responsible use of NGS and SNP microarrays in forensic contexts. One of the greatest challenges in forensic genomics is ensuring that data privacy, consent, and ethical use are upheld, especially in cases involving SNP-based inference of phenotypes or ancestry.

Policies must address:

- Informed consent for the use of genetic data in FIGG,
- Transparency in the secondary use of consumer genetic data,
- Legal admissibility of NGS-generated evidence,
- Proportionality in the use of FDP and ancestry prediction, and
- Protection of sensitive genetic information under legislation such as the GDPR and national data protection laws.

Collaborative efforts between forensic practitioners, policymakers, legal experts, and ethical advisory bodies are needed to develop a comprehensive regulatory framework. This will not only facilitate the safe and equitable implementation of advanced technologies but also ensure that the use of forensic genomics remains scientifically robust, socially responsible, and legally defensible.

## Conclusions

STR typing via CE remains the gold standard for forensic investigations. It excels in personal identification and kinship testing due to its rapid turnaround, cost-effectiveness, and well-established protocols, databases, and legal frameworks. However, despite the high polymorphism of STRs, their limited multiplexing capacity constrains their effectiveness in complex kinship analyses, especially beyond second-degree relationships, and in cases involving degraded samples or DNA mixtures. While the general lack of association between STRs and phenotypic traits is an ethical advantage, their inability to provide investigative leads in the absence of comparative profiles remains a limitation.

NGS addresses many of CE’s shortcomings. It allows for more detailed STR analysis through full-locus sequencing, enhancing discrimination power, mixture deconvolution, and the resolution of complex relationships. NGS also supports the use of smaller amplicons, which improves performance with degraded DNA. Furthermore, SNPs, due to their abundance, stability, and informativeness for traits such as ancestry and appearance, offer significant value as supplementary markers. Although SNPs individually have low polymorphism, large-scale panels can match or exceed the discrimination power of STRs. Their utility in distant kinship testing and phenotype prediction is becoming increasingly apparent. Moreover, new developments such as microhaplotypes and intensity-based mixture analysis further enhance their applicability. Despite these advantages, the uptake of NGS in forensic practice remains limited due to high costs, technical complexity, and the need for standardised interpretation protocols.

Similarly, DNA microarrays present a promising and more affordable alternative for SNP typing, particularly in long-range kinship analysis and forensic genetic genealogy. While challenges persist, notably in analysing degraded DNA and mixtures, emerging analytical methods show potential to overcome these limitations.

To move forward, forensic genetics must prioritise research, standardisation, and ethical governance. A key obstacle remains the lack of a comprehensive legal framework for newer genomic technologies, which hinders their routine adoption. However, the widespread use of NGS and microarrays in clinical and commercial settings suggests that affordability and accessibility will improve over time.

Change within forensic science is often met with resistance. Yet, just as STR profiling rose to prominence through decades of research and validation, so too can NGS and SNP microarrays establish themselves as indispensable tools. Their successful integration will depend on coordinated efforts among forensic scientists, legal professionals, and policymakers. A hybrid workflow, combining CE for routine cases, NGS for challenging samples, and SNP microarrays for extended kinship and investigative leads, offers the most pragmatic path forward. Alongside this, investment in bioinformatics training, the expansion of forensic DNA databases, and the development of robust legal and ethical guidelines are essential.

With continued research, validation, and collaboration, NGS and SNP microarrays can transition from emerging technologies to standard components of forensic casework, ultimately improving the scope, accuracy, and reliability of forensic investigations.

## Data Availability

No datasets were generated or analysed during the current study.

## Abbreviations

aiSNPs: Ancestry-informative SNPs
CE: Capillary electrophoresis
CODIS: Combined DNA index system
DTC: Direct-to-consumer
ENFSI: European network of forensic science institutes
ESS: European standard set
FBI: Federal bureau of investigation
FDP: Forensic DNA phenotyping
FIGG: Forensic investigative genetic genealogy
FORCE: FORensic capture enrichment
GDPR: General data protection regulation
GSA: Global screening array
iiSNPs: identity-informative SNPs
ISFG: International society for forensic genetics
ISO/IEC: International organization for standardization and international electrotechnical commission
MPS: Massively parallel sequencing
NDIS: National DNA index system
NDNAD: National DNA database
NGS: Next generation sequencing
PCR: Polymerase chain reaction
piSNPs: phenotype-informative SNPs
RFLP: Restriction fragment length polymorphism
RMP: Random match probability
SNP: Single nucleotide polymorphism
SNV: Single nucleotide variants
STR: Short tandem repeat
STRSeq: STR sequencing project
SWGDAM: Scientific working group DNA analysis methods
TH: Tyrosine hydroxylase
VISAGE: Visible attributes through genomics
VNTR: Variable number tandem repeat
X-STRs: X-chromosomal STRs
Y-SNPs: Y-chromosomal SNPs
Y-STRs: Y-chromosomal STRs
